# A New Antiproliferative and Antioxidant Peptide Isolated from *Arca subcrenata*

**DOI:** 10.3390/md11061800

**Published:** 2013-05-24

**Authors:** Lili Chen, Liyan Song, Tingfei Li, Jianhua Zhu, Jian Xu, Qin Zheng, Rongmin Yu

**Affiliations:** 1 Biotechnological Institute of Chinese Materia Medica, Jinan University, Guangzhou 510632, China; E-Mails: chennybest@163.com (L.C.); tzhujh@jnu.edu.cn (J.Z.); lenovo1688@126.com (J.X.); 2 College of Pharmacy, Jinan University, Guangzhou 510632, China; E-Mail: zhengqin027@163.com; 3 Guangdong Food and Drug Vocational-Technical School, Guangzhou 510663, China; E-Mail: phoebe2326@126.com

**Keywords:** *Arca subcrenata*, peptide, antiproliferative activity, antioxidant activity

## Abstract

A new antitumor and antioxidant peptide (H3) was isolated from *Arca subcrenata* Lischke using ion exchange and hydrophobic column chromatography. The purity of H3 was over 99.3% in reversed phase-high performance liquid chromatography (RP-HPLC) and the molecular weight was determined to be 20,491.0 Da by electrospray-ionization mass spectrometry (ESI-MS/MS). The isoelectric point of H3 was measured to be 6.65 by isoelectric focusing-polyacrylamide gel electrophoresis. Partial amino acid sequence of this peptide was determined as ISMEDVEESRKNGMHSIDVNHDGKHRAYWADNTYLM-KCMDLPYDVLDTGGKDRSSDKNTDLVDLFELDMVPDRKNNECMNMIMDVIDTN-TAARPYYCSLDVNHDGAGLSMEDVEEDK via MALDI-TOF/TOF-MS and *de novo* sequencing. The *in vitro* antitumor activity of H3 was evaluated by 3-(4,5-dimethyl-2-thiazolyl)-2,5-diphenyl-2*H*-tetrazolium bromide (MTT) assay. The result indicated that H3 exhibited significant antiproliferative activity against HeLa, HepG2 and HT-29 cell lines with IC_50_ values of 10.8, 10.1 and 10.5 μg/mL. The scavenging percentage of H3 at 8 mg/mL to 2,2-diphenyl-1-picrylhydrazyl (DPPH) and hydroxyl radicals were 56.8% and 47.5%, respectively.

## 1. Introduction

Cancer is the leading cause of death in developed countries and the second in developing countries. The global economic burden for this disease continues to increase heavily because of the aging and growth of the world population alongside an increasing adoption of cancer-causing behaviors. On the basis of GLOBOCAN 2008, about 12.7 million cancer cases and 7.6 million cancer deaths occurred in 2008 [1]. Cervical cancer currently ranks as the second most common cause of cancer-related morbidity and the third most common cause of mortality worldwide [2]. Hepatocellular carcinoma is the major histological subtype among liver cancer. Among the top causes of cancer deaths, hepatocellular carcinoma is especially prevalent in parts of Asia and Africa [3]. Colorectal cancer is the third most diagnosed cancer and has a mortality rate of nearly one in two [4]. Many colorectal cancers are deemed to arise from adenomatous polyps in the colon [5]. However, the present cancer treatments such as the use of chemotherapeutic agents, radiation, and surgery have not been effective in reducing the currently high mortality rate for most forms of cancer [6]. Therefore, it is necessary to explore new drugs/agents to prevent and treat cancers.

Marine animals represent an essentially prospective resource for the discovery and development of potential antitumor drugs because they live in very exigent, competitive and aggressive surroundings, which are very different in many aspects from the land. The diverse sequences of peptides may be due to adaptation to the special environment [7]. Recently, an increasing number of investigations have focused on bioactive peptides in the sea. Many bioactive peptides with anticancer potential have been extracted from various marine animals like sponges, tunicates, soft corals, nudibranchs, sea hares, bryozoans, sea slugs and other marine organisms [8]. More than ten new experimental antitumor agents derived from marine sources have entered clinical trials, including dolastatin 10 [9], kahalalide F [10], aplidine [11], Yaku’amides A and B [12].

*Arca subcrenata* Lischke is a marine invertebrate that belongs to Arcidae family under Phylum Mollusca, Class Lamellibranchiata. This marine organism is widely distributed in China, Japan, Korea, *etc.* and has been used in treatments of blood deficiency, epigastric pain and indigestion for centuries in Chinese Traditional Medicines [13]. The studies on the content of proteins, proportion of amino acids, ultraviolet spectrum analysis and trace element analysis of this species have been previously reported [14]. Its hydrolysate was also reported to have hypoglycemic and hypolipidic activities on mice [15]. Two proteins, G6 and G-4-2, were isolated from *A. subcrenata* and antioxidant activities of hydrolysates of *A. subcrenata* prepared with three proteases were also investigated by our research group [16,17]. As a continuous work to look for more efficient bioactive peptides from *A. subcrenata*, we report a novel antitumor and antioxidant peptide compound in the present paper.

## 2. Results

### 2.1. Bioassay-Guided Isolation *in Vitro*

The total proteins of *A. subcrenata* were subjected to the antiproliferative assay against cancer cells. The results showed that the total proteins suppressed the proliferation of HeLa, HepG2 and HT-29 cells with IC_50_ values of 106.5, 264.8 and 315.4 μg/mL, respectively (Table 1). The total proteins were fractionated by salting-out at increasing saturation levels of ammonium sulfate. Fraction I, Fraction II, and Fraction III were obtained at the various ammonium sulfate saturation. Fraction III exhibited significant inhibition on the proliferation of HeLa, HepG2 and HT-29 cells with IC_50_ values of 85.3, 83.7 and 92.5 μg/mL, respectively (Table 1). The results suggested that Fraction III warranted further purification in order to obtain purified active peptides.

**Table 1:** Antiproliferative action of protein samples against five cancer cell lines (IC_50_ μg/mL ± SD, *n =* 3).

Samples	IC_50_ (μg/mL)				
	HeLa	A549	HepG2	HT-29	SPC-A-1
Total proteins	106.5 ± 13.2	836.2 ± 76.3	264.8 ± 20.6	315.4 ± 25.3	635.8 ± 46.5
Fraction I	305.3 ± 24.1	1089.6 ± 85.4	316.8 ± 20.9	325.7 ± 19.8	587.4 ± 30.6
Fraction II	265.5 ± 17.2	813.7 ± 80.4	274.3 ± 22.6	295.9 ± 18.1	567.5 ± 34.3
Fraction III	85.3 ± 7.3	789.4 ± 75.6	83.7 ± 5.6	92.5 ± 8.2	314.4 ± 28.5
Fraction P1	312.6 ± 26.8	632.5 ± 83.1	214.5 ± 11.4	255.4 ± 14.8	468.2 ± 28.6
Fraction P2	11.4 ± 3.1	232.2 ± 14.6	10.4 ± 2.7	13.0 ± 3.3	727.6 ± 67.9
Fraction P3	231.7 ± 14.3	547.4 ± 27.8	275.4 ± 15.6	258.5 ± 21.5	889.2 ± 86.7
Fraction P4	327.2 ± 19.1	778.6 ± 70.8	253.8 ± 12.6	308.2 ± 25.4	845.3 ± 83.9
H1	146.7 ± 13.6	nd	132.2 ± 11.5	135.3 ± 12.5	nd
H2	133.6 ± 12.3	nd	120.7 ± 10.8	125.7 ± 11.3	nd
H3	10.8 ± 2.8	nd	10.1 ± 2.3	10.5 ± 2.4	nd
Cisplatin	1.13 ± 0.06	0.87 ± 0.03	0.98 ± 0.04	0.94 ± 0.04	2.32 ± 0.07

Fraction I: The precipitate after 0%–35% (NH_4_)_2_SO_4_ saturation; Fraction II: The precipitate after 35%–75% (NH_4_)_2_SO_4_ saturation; Fraction III: The precipitate after 70%–100% (NH_4_)_2_SO_4_ saturation; nd: not detected.

**Figure 1:**
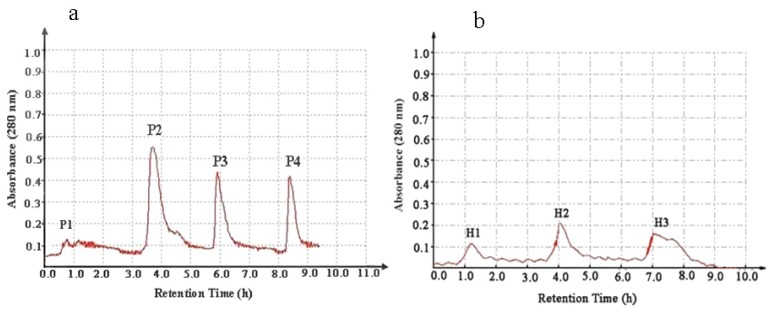
Purification of the antitumor peptide from *A. subcrenata* crude protein. (**a**) Separation of antitumor peptides by diethylaminoethanol (DEAE) sepharose fast flow anion exchange chromatography; (**b**) Purification of Fraction P2 using phenyl sepharose CL-4B hydrophobic chromatography.

The lyophilized Fraction III was loaded onto a diethylaminoethanol (DEAE) sepharose fast flow anion exchange column resulting in four peaks, named P1–P4 (Figure 1a). As shown in Table 1, Fraction P2 possessed the strongest antitumor action and was further purified with a Phenyl Sepharose CL-4B hydrophobic chromatography column. As shown in Figure 1b, three peptides were isolated and named consecutively H1–H3. H3 exhibited the highest antiproliferative activity with an IC_50_ of 10.8, 10.1, and 10.5 μg/mL against HeLa, HepG2 and HT-29 cell lines, respectively.

**Figure 2:**
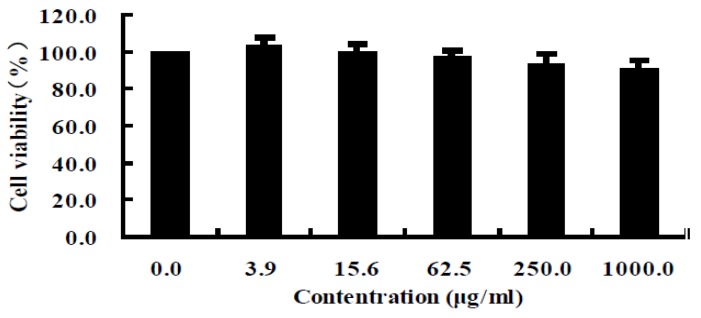
Cytotoxicity of H3 against the L-02 cell line. Data are presented as mean ± SD of three independent experiments.

The cytotoxic effect of H3 was tested the using L-02 cell line and the results showed no toxicity at various concentrations ranging from 3.9 to 1000 μg/mL (Figure 2). The cell viability data confirmed that Fraction H3 was noncytotoxic.

### 2.2. Characterization of Purified Peptide

**Figure 3:**
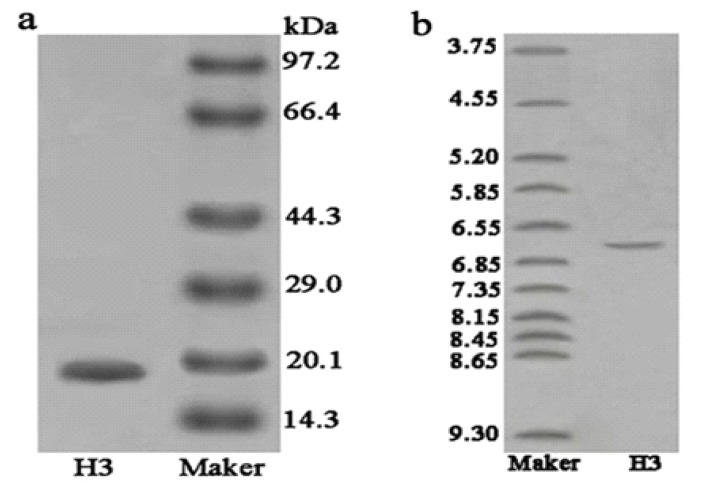
Electrophoresis. Purity and molecular mass analysis of H3 by sodium dodecyl sulfate polyacrylamide gel electrophoresis (SDS-PAGE): (**a**) Lane 1, H3; Lane 2, molecular weight marker; (**b**) Isoelectric point determination of H3 by isoelectric focusing polyacrylamide gel electrophoresis (IEF-PAGE): Lane 1, PI marker; Lane 2, H3.

**Figure 4:**
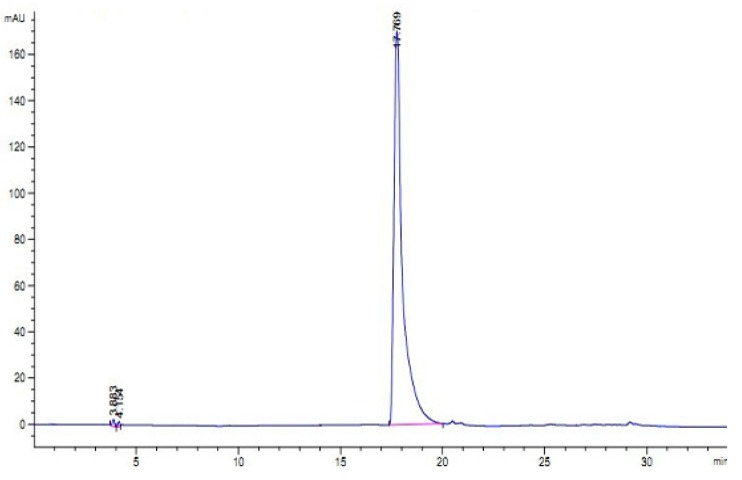
Reversed phase-high performance liquid chromatography (RP-HPLC) profile of H3. Performed on an Agilent 1100 HPLC system fitted with a ZORBAX^®^ 300SB-C8, Agilent column (5 μm, 300 Å, 4.6 × 250 mm).

**Figure 5:**
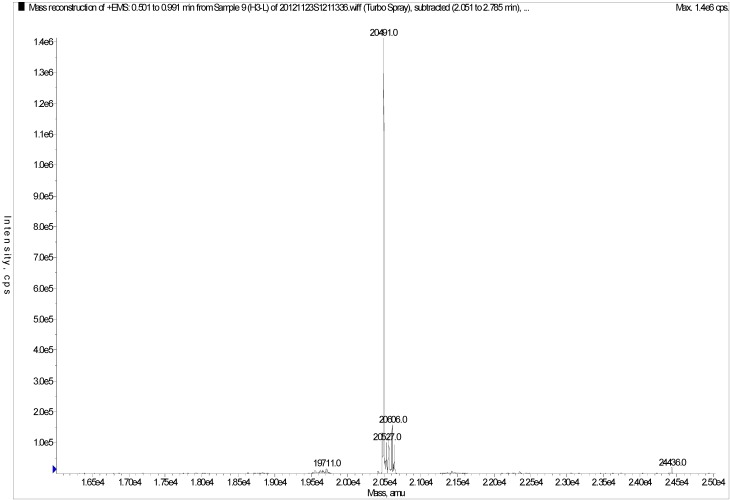
Mass spectrum of H3.

As shown in Figure 3a, H3 gave a single band in sodium dodecyl sulfate polyacrylamide gel electrophoresis (SDS-PAGE), indicating that it was electrophoretically homogeneous. Furthermore, the results of RP-HPLC (Figure 4) showed that the purity of H3 was 99.3%. This information also indicated that H3 was a homogeneous peptide. The molecular weight of H3 was estimated by SDS-PAGE, with molecular weight markers ranging from 14.3 to 97.2 kDa as standard. According to the calibration curve, the molecular weight of H3 was approximately 20.0 kDa (Figure 3a). The isoelectric focusing polyacrylamide gel electrophoresis (IEF-PAGE) showed that the isoelectric point of peptide H3 was 6.65 (Figure 3b). To determine the precise molecular mass, H3 was loaded onto electrospray-ionization mass spectrometry (ESI-MS/MS). The result showed that the accurate molecular weight was 20,491.0 Da (Figure 5).

### 2.3. *De Novo* Sequencing of Peptide

Matrix-Assisted-Laser-Desorption/Ionization-Time-of-Flight-Mass-Spectrometry (MALDI-TOF/TOF-MS) of H3 was measured with different ion signals in the mass range of 1200–2800 Da. The seven precursor ions (*m*/*z* 1210.55, *m*/*z* 1567.74, *m*/*z* 1684.77, *m*/*z* 1870.87, *m*/*z* 2325.05, *m*/*z* 2373.05, *m*/*z* 2759.26) were further detected by MS/MS analysis. Seven MS/MS spectra consisting of a series of y and b ions and several a ions were obtained and used for *de novo* sequencing. Based on the manual calculation of the molecular weights and the *m*/*z* values, the amino acid sequence for each peptide was determined. The sequence of fragment ion *m*/*z* 1210.55 was ISMEDVEESR, fragment *m*/*z* 1567.74 sequence was KNGMHSIDVNHDGK, fragment *m*/*z* 1684.77 with the amino acid sequence: HRAYWADNTYLMK, fragment *m*/*z* 1870.88 with the amino acid sequence: CMDLPYDVLDTGGKDR, fragment m/z 2325.05 with the amino acid sequence: SSDKNTDLVDLFELDMVPDR, fragment *m*/*z* 2373.05 with the amino acid sequence: KNNECMNMIMDVIDTNTAAR, fragment *m*/*z* 2759.26 with the amino acid sequence: PYYCSLDVNHDGAGLSMEDVEEDK. After assembling sequences of the seven fragmentations, the peptide partial sequence gave as ISMEDVEESRKNGMHSIDVNHDGKHRAYWADNTYLMKCMD-LPYDVLDTGGKDRSSDKNTDLVDLFELDMVPDRKNNECMNMIMDVIDTNTAARPYYCSLD-VNHDGAGLSMEDVEEDK. The amino acid sequence alignment was performed against the National Center for Biotechnology Information Basic Local Alignment Search Tool (NCBI BLAST) database and the low similarity of the amino acid sequences indicated that H3 was a novel peptide.

### 2.4. *In Vitro* Antioxidant Activity

**Figure 6:**
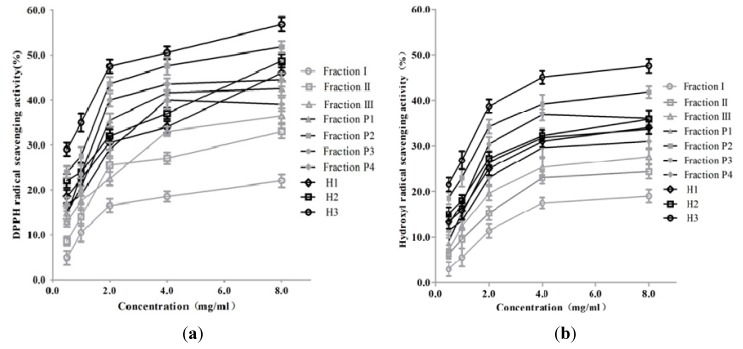
DPPH (**a**) and hydroxyl (**b**) radical-scavenging activities of fractions from ammonium sulfate precipitation, ion exchange chromatography and further hydrophobic chromatography. Data are presented as mean ± SD of three independent experiments.

As shown in Figure 6, all fractions were capable of scavenging both DPPH and hydroxyl radicals *in vitro* in a dose-dependent manner. H3 exhibited the highest scavenging activities against DPPH and hydroxyl radicals with values of 56.8% and 47.5%, respectively. And the value of the positive control reached 96.8% and 93.6%, respectively, at the concentration of 1.0 mg/mL. The result demonstrated that the peptides isolated from *A. subcrenata* presented antioxidant activity with higher activity in quenching DPPH over hydroxyl radical.

## Discussion

Marine organisms are an immense source of new biologically active compounds. One of the approaches for the effective acquisition of bioactive peptides from marine organisms is direct extraction, which is widely applied to improve and upgrade the functional and nutritional properties of proteins [18]. 

In the current study, a peptide with significant antiproliferative activity and high antioxidant property was isolated from *A. subcrenata*. As all extraction and separation procedures were conducted at the low temperature, it was possible to obtain natural product peptides [19,20]. All the fractions were tested for their antiproliferative activity by MTT assay. As shown in Table 1, H3 exhibited the strongest antiproliferative effect against HeLa, HepG2 and HT-29 cell lines in the fractions tested. The antitumor activity of this new peptide showed an about 30-fold increase over the crude extract and was stronger than that described in our previous report [16]. The isolated peptide exerted more potent antiproliferative activity (IC_50_ = 10.1 μg/mL) towards HepG2 than the peptide isolated from *Nemipterus japonicus* backbone (IC_50_ = 61.1 μg/mL) [21]. The L-02 cell line was selected in our experiment to evaluate cytotoxicity [22,23] of H3 and the result showed that it was non-toxic till 1000 μg/mL. It demonstrated that H3 possessed remarkable antitumor effects against several cancer cells without cytotoxicity.

The MS result indicated that the size of H3 was comparatively bigger than that previously purified from *A. subcrenata* [16], but smaller in size than the antitumor peptide from *Syngnathus acus* [24]. However, the *in vitro* antitumor activity of H3 was better than that of the other proteins. It seems that the antiproliferative activity of H3 might not be directly related to the molecular weight. 

Sequence information of H3 was obtained by MALDI-TOF/TOF mass spectrometer. The amino acid sequence of H3 had a slight similarity to other peptides from the BLAST search of NCBI data base. Thus, H3 could be proposed as a novel antiproliferative peptide. Apparently, the purified peptide contained both essential and non-essential amino acids. The presence of aromatic amino acids like tyrosine allowed direct electron transfer to reactive oxygen species (ROS) [25]. In addition, H3 was equipped with potent amino acids like cysteine, alanine, phenylalanine, glycine and histidine, which were known to enhance the antioxidant activity [26,27]. This might be the reason why H3 shows a higher scavenging ability against DPPH and hydroxyl radicals than other fractions. 

On the other hand, all fractions in our experiment were tested for DPPH and hydroxyl radical quenching efficiency. The increase of antioxidant activity always accompanied every isolation step. Likewise, H3 demonstrated greater antiprolifertative activity than the other fractions towards HeLa, HepG2 and HT-29 cancer cells. The results suggested that the antioxidant and antiproliferative activities of the peptide were interrelated. ROS, including DPPH and hydroxyl radicals, are unstable and react readily with other groups or substances in the body, resulting in cell damage, which is believed to induce human disease such as diabetes mellitus, cancer, neurodegenerative, inflammatory and Alzheimer’s disease [28,29,30]. Therefore, it might be proposed that higher antioxidant activity leads to better antitumor activity [31]. This conclusion was also consistent with reports in which antioxidants had the potential to prevent and treat diseases associated with active oxygen species, especially some forms of cancer [32,33]. As reported by Chinery, antioxidants can enhance the cytotoxicity of chemotherapeutic agents in colorectal cancer [34]. Recently, investigators reported that the oxygen radical scavenging capacity of most protein fractions was correlated (*R*^2^ > 0.86, *p* < 0.01) with the antiproliferative activity against Caco-2 colon cancer, HepG2 liver cancer and MCF-7 breast cancer cell lines [3].

## 4. Experimental Section

### 4.1. Materials

DEAE Sepharose Fast Flow and Phenyl Sepharose CL-4B were purchased from GE Healthcare. Tris, SDS, MTT and Cisplatin were obtained from Sigma Chemical Co. (St. Louis, MO, USA). Ascorbic acid (vitamin C, Vc), hydrogen peroxide (H_2_O_2_) and ferrous sulfate (FeSO_4_) were obtained from Guangzhou Chemical Reagent Co., Guangzhou, China. Other commercially available chemicals and reagents were of analytical grade.

### 4.2. Sample Collection

The samples of *A. subcrenata* were collected from Huangsha seafood market, Guangzhou, China. All of them were identified by Rongmin Yu (Jinan University, Shandong, China). The visceral mass was dissected from *A. subcrenata*, weighed and stored at −20 °C until used.

### 4.3. Preparation of Crude Protein

All extraction and separation procedures were carried out at 4 °C. The visceral mass (200 g) of *A. subcrenata* was washed with distilled water and extracted with 600 mL of phosphate buffer, then a KQ-250B ultrasonic cleaner (Shanghai Yuzhen Co., Shanghai, China) with straight probe and continuous pulse was used to ultrasound for 40 min. After centrifugation (10,000× *g*, 30 min), the supernatant was collected as total protein and then was fractionated by salting-out with increasing concentrations of ammonium sulfate. Three fractions were prepared with various saturated ammonium sulfate solutions. Every protein pellet was dissolved in a small amount of 30 mM Tris-HCl buffer (pH 8.0) and dialyzed against distilled water to completely remove any residual ammonium sulfate [35]. The dialyzed solution was then lyophilized for further usage.

The dialyzed solution was then lyophilized for further usage.

### 4.4. *In Vitro* Antiproliferative Assay

#### 4.4.1. Cell Lines and Culture Conditions

Cervix epithelioid carcinoma (HeLa), lung adenocarcinoma cell lines (A549, SPC-A-1), human liver carcinoma (HepG2), colon adenocarcinoma (HT-29) and normal human liver cells (L-02) were provided by the School of Life Sciences, Sun Yat-sen University, Guangzhou, China. All cells were cultured in Dulbecco’s modified eagle minimum essential medium (DMEM) medium supplemented with 100 mg/mL streptomycin containing 10% fetal bovine serum (FBS) and 100 units/mL penicillin. Cells were incubated at 37 °C in a humidified atmosphere with 5% (v/v) CO_2_.

#### 4.4.2. Cell Antiproliferative Assay

Evaluation of cancer cell growth inhibition was assessed by the MTT assay [36]. Exponentially growing cancer cells were seeded into 96-well culture plates (4 × 10^3^ cells/well) and incubated at 37 °C in a humidified incubator with 5% CO_2_ for 24 h. Cells were incubated with different concentrations of sample for another 72 h and cisplatin was used as the positive control. Untreated cells were used as negative control. Then, 20 μL of 5 mg/mL MTT was added to each well and the plates were incubated for 4 h. After the removal of MTT, the precipitate was solubilized in dimethyl sulfoxide (DMSO) (100 μL/well) and the absorbance was measured on a microplate reader (Bio-Rad, Hercules, CA, USA) at a wavelength of 570 nm. The concentration of the sample that caused 50% growth inhibition was referred to as the IC_50_ value.

#### 4.4.3. Cytotoxicity Assay

The cytotoxicity assay was similarly conducted according to the method of the MTT assay. L-02 cell lines were used to examine the cytotoxic effect of the peptide. The experiment was performed as described above. The percentage of cytotoxicity was calculated as follows:

Cytotoxicity (%) = (1 − *A*_570_ of experimental well)/*A*_570_ of control well

### 4.5. Purification of Antiproliferative Peptide

#### 4.5.1. Anion Exchange Chromatography

The lyophilized *A. subcrenata* viscera protein (10 mg/mL) was dissolved in 30 mM Tris-HCl buffer (pH 8.0), and loaded onto a DEAE Sepharose Fast Flow anion exchange column (2.5 cm × 40 cm) equilibrated with the above Tris-HCl buffer. The column was stepwise eluted with 0, 0.1, 0.3 and 2 M NaCl prepared in the same buffer at a flow rate of 1 mL/min. Each fraction was collected at a volume of 5 mL and was monitored at 280 nm. Fractions were then concentrated and lyophilized and antitumor activities were evaluated. The fraction with the strongest antiproliferative activity was subjected to the next separation. 

#### 4.5.2. Hydrophobic Chromatography

The sample was dissolved in 1.5 M (NH_4_)_2_SO_4_ prepared with 30 mM phosphate buffer (pH 8.0) and loaded onto a Phenyl Sepharose CL-4B hydrophobic chromatography column (2.5 cm × 40 cm) which had previously been equilibrated with the above buffer. A stepwise elution was carried out with decreasing concentrations of (NH_4_)_2_SO_4_ (1.5, 1.0 and 0 M) dissolved in 30 mM phosphate buffer (pH 8.0) at a flow rate of 1 mL/min. Each fraction was collected at a volume of 5 mL and was monitored at 280 nm. Fractions were then lyophilized and antitumor activities were determined. The fraction having the strongest antiproliferative activity was collected and used for further experiments

### 4.6. Characterization of Antiproliferative Peptide

#### 4.6.1. Protein Determination

Protein concentration was measured according to the reference [37]. Bovine serum albumin (BSA) was used as standard protein.

#### 4.6.2. Sodium Dodecyl Sulfate Polyacrylamide Gel Electrophoresis (SDS-PAGE)

The fractions of eluted peptides were analyzed by SDS-PAGE [38], using an acrylamide concentration of 5% for the stacking gel and 16% for the running gel. SDS-PAGE analysis under reducing conditions was used to check the purity of peptide and determine molecular weight. Protein bands were detected by the coomassie blue staining method [39]. 

#### 4.6.3. Isoelectric Focusing-Polyacrylamide Gel Electrophoresis (IEF)

Isoelectric point of protein sample was determined by immobilized pH gradient (IPG) isoelectric focusing-polyacrylamide gel electrophoresis (IEF-PAGE). The gel was made with 40% ampholine at a pH range from 3.5 to 10.0 and stained with coomassie brilliant blue R-250 [40].

#### 4.6.4. Reversed Phase-High Performance Liquid Chromatography (RP-HPLC)

HPLC analyses were performed on an Agilent 1100 HPLC system fitted with a ZORBAX^®^ 300SB-C8, Agilent column (5 μm, 300 Å, 4.6 mm × 250 mm). The elution solvent system was composed of water-trifluoroacetic acid (solvent A; 100:0.1, v/v) and acetonitrile-trifluoroacetic acid (solvent B; 100:0.1, v/v). The peptide was separated using a gradient elution from 30% to 45% of solvent B for 30 min at a flow rate of 1.0 mL/min. Detection wavelength was set at 280 nm and column temperature was 25 °C.

#### 4.6.5. Molecular Mass Determination

To determine the precise molecular weight of the purified peptide, the sample was dissolved in water of HPLC grade and loaded into an API type 4000 QTRAP mass spectrometer (Applied Biosystem, Foster City, CA, USA). The sample was passed at a flow rate of 20 μL/min via the electro spray interface, which was operated in the positive electrospray ionization (ESI + ve) mode. The gas used for drying (35 psi) and ESI nebulization (45 psi) was high-purity nitrogen. Spectra were recorded over the mass/charge (*m*/*z*) range 10,000–25,000.

### 4.7. Identification of Peptide by MALDI-TOF/TOF-MS

The amino acid sequence of peptide was identified by the reference [41] with some modifications. H3 was excised from SDS-PAGE gel and digested. Tryptic peptides were lyophilized and dissolved in 10 μL of a 50% acetonitrile/0.1% TFA solution. An amount of 0.4 μL of the sample was spotted onto the MALDI sample target plate, and then 0.4 μL of a saturated matrix solution of α-cyano-4-hydroxycinnamic acid prepared in 50% acetonitrile/0.1% TFA was added. Peptide mass spectra were obtained on a 4800 Proteomics Analyzer MALDI-TOF/TOF mass spectrometer (Applied Biosystems, Foster City, CA, USA) in the positive ion reflection mode. After an external calibration with a mixture of angiotensin II (Mr, 1046.54180), angiotensin I (Mr, 1296.68478), substance P (Mr, 1347.73543), bombesin (Mr, 1619.82235), ACTH clip 1–17 (Mr, 2093.0868), ACTH clip 18–39 (Mr, 2465.1990), Somatostatin 28 (Mr, 3147.4714), spectra were obtained in the mass range between 900 and 3500 Da with 500 laser shots. For each sample spot, a data dependent acquisition method was created to select the seven most intense peaks for subsequent MS/MS data acquisition, excluding those from the matrix, due to trypsin autolysis or acrylamide peaks. MS/MS spectra were acquired with 1200 laser shots in the mass range from 10 Da to the mass of parent ion using an interpretation method presented on instrument software, where the seven most intense peaks were selected and MS/MS spectra were generated automatically. To ensure a reliable identification, the results from both the MS and MS/MS spectra were used in the database search. Peptide identification was accepted when the score read by the Mascot search routine was higher than 90. The sequence of peptide fragments was determined by *de novo* sequencing using the Applied Biosystems software as presented by Yergey [42].

### 4.8. Determination of *in Vitro* Antioxidant Activities

#### 4.8.1. DPPH Radical Scavenging Activity

One hundred and ninety micro liters DPPH of ethanol solution (0.2 mM) was added to 10 μL of sample solution of gradient concentrations (0.5, 1.0, 2.0, 4.0, 8.0 mg/mL). Vc was used as the positive control with final concentrations of 0.1, 0.2, 0.4, 0.8 and 1.0 mg/mL. The absorbance was measured at 517 nm after incubating at 37 °C for 30 min. The lower absorbance of the reaction mixture indicated the higher free radical scavenging activity. DPPH radical scavenging ability was calculated using the following equation:

DPPH radical scavenging ability (%) =((*A*_0_ − *A*_1_)/*A*_0_) × 100

where *A*_0_ was the absorbance of control (without sample) and *A*_1_ was the absorbance in the presence of sample [43]. 

#### 4.8.2. Hydroxyl Radical Scavenging Activity

The hydroxyl radical scavenging assay was evaluated using the hydroxyl radical system generated by the Fenton reaction. Samples were dissolved in distilled water with final concentrations of 0.5, 1.0, 2.0, 4.0, 8.0 mg/mL. Vc was used as the positive control. The reaction mixture contained 30 μL of 1,10-phenanthroline (5.0 mM), 30 μL of FeSO_4_ (5.0 mM), 100 μL of distilled water and 20 μL of sodium phosphate buffer (0.1 M, pH 7.4). Then 10 μL of sample and 10 μL of H_2_O_2_ (0.1%) were added. After incubation at room temperature for 60 min, the absorbance of the mixture was measured at 510 nm. The hydroxyl radical scavenging activity was calculated according to the equation:

Hydroxyl radical scavenging activity (%) = (*A*_s_ − *A*_0_)/(*A*_c_ − *A*_0_) × 100

where *A*_s_ was the absorbance of the sample, *A*_0_ was the absorbance of the blank solution, and *A*_c_ was the absorbance of the control solution [44].

### 4.9. Statistical Analysis

All of the tests were conducted in triplicate, and the experimental data were expressed as the mean ± standard deviation. GraphPad Prism 5.0 was used for statistical analysis. Statistical significance was determined using an unpaired, two-tailed Student’s *t*-test, with a 95% confidence interval.

## 5. Conclusions

A novel peptide isolated from *A. subcrenata* exhibiting significant antiproliferative activity against HepG2, HeLa and HT-29 cancer cells without cytotoxicity. And it showed high antioxidant activity against the radical scavenging efficiency on DPPH and hydroxyl radicals. The present report demonstrates that the marine peptide may act as one of the most potential sources of anticancer agents and will be attracting more and more attention in the research and development of new drugs.
